# Cutaneous Syncytial Myoepithelioma with Positive CD34 Immunohistochemical Staining: An Unusual Tumor and a Challenging Diagnosis

**DOI:** 10.3390/dermatopathology10030034

**Published:** 2023-08-30

**Authors:** Cesare Ariasi, Carola Romanò, Iacopo Ghini, Gaetano Licata, Luca Rubelli, Grazia Linda Artelli, Piergiacomo Calzavara-Pinton, Mariachiara Arisi

**Affiliations:** 1 Department of Dermatology, University of Brescia, 25123 Brescia, Italy; 2 Department of Pathology, University of Brescia, 25123 Brescia, Italy; 3 Dermatology Unit, San Antonio Abate Hospital, 91016 Trapani, Italy

**Keywords:** cutaneous syncytial myoepithelioma, skin neoplasms, EWSR1, CD34

## Abstract

Cutaneous syncytial myoepithelioma (CSM) is a rare type of cutaneous neoplasm that typically presents as a solitary and well-circumscribed nodule on the skin. It predominantly occurs on the upper and lower extremities of adult patients. Immunohistochemically, CSM is characterized by the co-expression of smooth muscle and epithelial markers. Fluorescence in situ hybridization (FISH) targeting the EWSR1 gene rearrangement is an important diagnostic tool for CSM. In our case report, we found the focal positivity for CD34, which has never been previously observed; this was mostly confined to a central area of the neoplasm.

## 1. Introduction

Myoepithelial tumors are most commonly identified in the salivary glands, encompassing mixed tumors (pleomorphic adenomas), myoepitheliomas, and myoepithelial carcinomas. However, they have also been reported in other anatomical locations such as the sinonasal tract, larynx, and lung. In recent years, similar morphological lesions arising primarily in soft tissues and bones have been documented. Additionally, the recognition of cutaneous myoepitheliomas has emerged, categorizing them within the spectrum of cutaneous neoplasms displaying myoepithelial differentiation, alongside “chondroid syringomas” (mixed tumors) [1].

The cutaneous syncytial myoepithelioma (CSM) is a rare cutaneous neoplasm belonging to the larger group of myoepithelial tumors. It was first described in 2004 by Hornick and Fletcher [2] and subsequently clinicopathologically and molecularly characterized in 2013 by Jo et al. [3]. CSM is histologically defined by an intradermal solid proliferation of spindle to ovoid cells, displaying distinct histopathological and molecular features. At the immunohistochemistry level, it infrequently shows keratin staining and on the molecular level, it frequently presents EWSR1 gene rearrangements, differing from standard myoepithelioma [4]. Typically, it presents as a solitary, well-circumscribed nodule on the skin, with sizes ranging from 3 to 27 mm (median of 8 mm) [3]. It predominantly occurs on the upper and lower extremities of adult patients, showing a slight male predilection (M:F = 2.5:1). The mean age of presentation is 39 years, although it has also been observed across a wide age range, spanning from 2 months to 74 years [4].

## 2. Case Presentation

**Figure 1:**
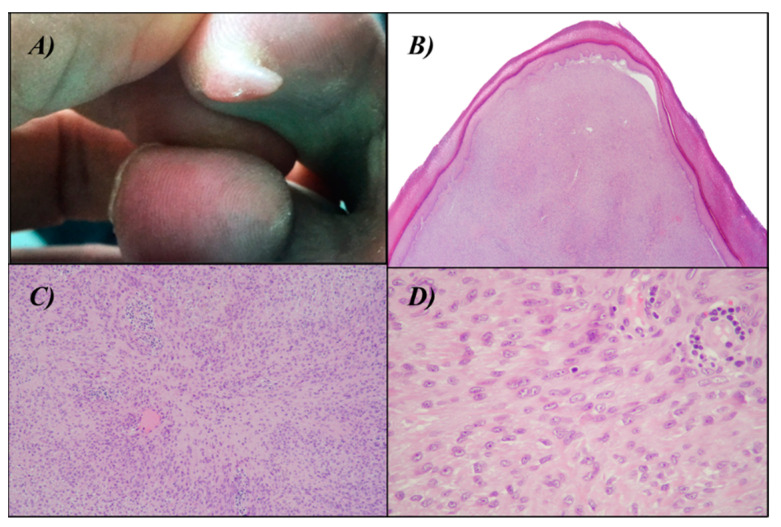
(**A**) raised white, painful, well-circumscribed nodule on the second toe of the right foot. (**B**) Histopathological image of the lesion (hematoxylin-eosin stain, 20× magnification): solid-growing lesion occupying the full-thickness dermis, surmounted with normotrophic epidermis with hyper-orthokeratosis. (**C**,**D**) Histopathological image of the lesion (hematoxylin-eosin stain, 100× and 400× magnification): small uniformly sized cells with spindled or histiocytoid or epitheliod morphology with palely eosinophilic syncytial cytoplasm.

We present the case of a 54-year-old Caucasian man with no significant medical history, who was referred to our outpatient dermatology clinic with a raised, painful, well-circumscribed nodule on the plantar surface of the second toe of his right foot, measuring approximately 10 × 7 mm (Figure 1A). The patient experienced significant discomfort and functional limitation due to the lesion, so we performed a local surgical excision with close margins to address the symptoms and functional impairment caused by the nodule.

A histological examination revealed a papular-shaped intradermal nodule with a solid growth pattern and indistinct tumor borders, involving the deep surgical margin. The neoplastic cells within the nodule displayed a uniform size and exhibited small nuclei with an ovoid to histiocytoid shape, with inconspicuous nucleoli. The cytoplasm of these cells appeared palely eosinophilic and indistinct. They were arranged in a haphazard to fascicular growth pattern, maintaining the integrity of the epidermis and the eccrine ducts. Mitotic figures were exceedingly rare, ranging from 0 to 1 per 10 high-power field (HPF). The proliferation index, as indicated by Ki-67, was estimated to be around 5% (Figure 1B–D).

**Figure 2:**
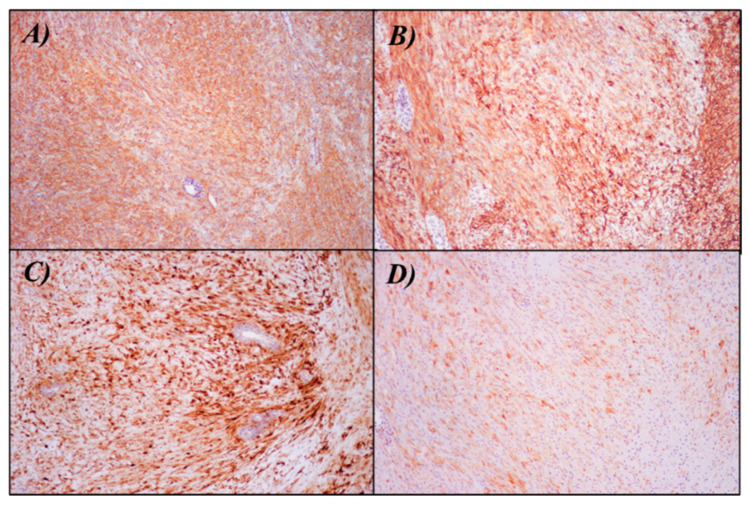
Immunohistochemistry showing positivity for (**A**) SMA (100× magnification), (**B**) EMA (100× magnification), (**C**) S-100 (100× magnification), and (**D**) Calponin (100× magnification).

**Figure 3:**
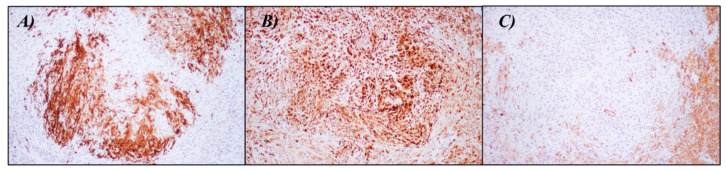
Immunohistochemistry showing the central area of the neoplasm with positivity for (**A**) CD34 (100× magnification), (**B**) S100 (100× magnification), and negativity for (**C**) SMA (100× magnification).

**Figure 4:**
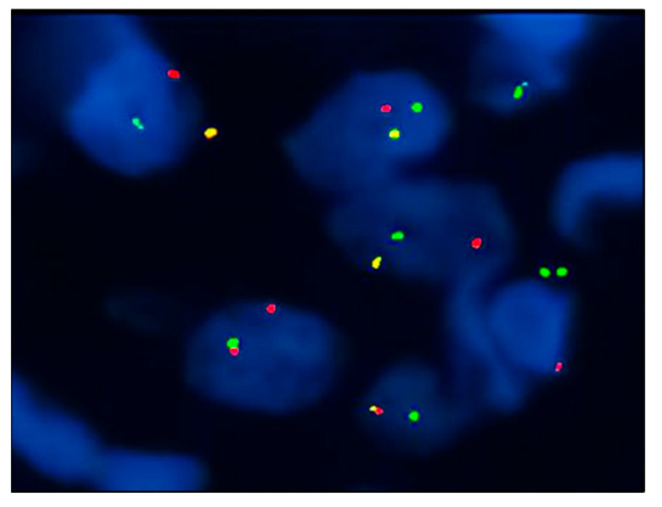
EWSR1 break-apart FISH: the presence of split red and green signals indicates gene rearrangement.

The neoplastic cells demonstrated a myoepithelial phenotype with diffuse positivity for S100, Epithelial Membrane Antigen (EMA), Smooth Muscle Actin (SMA), and focally for Calponin (Figure 2). They were consistently negative for SOX10, Melan-A, HMB-45, Desmin, CD31, and ERG. INI1 expression was retained. Interestingly, a central area of the tumor showed a focal and staining for CD34 (Figure 3). Based on these features, an initial hypothesis of cutaneous syncytial myoepithelioma was considered. To confirm the diagnosis, we demonstrated the rearrangement of the EWSR1 gene with a fluorescence in situ hybridization (FISH) test (Vysis LSI EWSR1 22q12 Dual Color Break Apart Rearrangement FISH Probe Kit-Abbott) (Figure 4). A few months later, further surgery to achieve free margins was performed.

## 3. Discussion

To the best of our knowledge, less than 80 cases have been previously documented in the literature, making this tumor a very rare entity [2,3,4,5,6,7]. CSM from a clinical perspective does not display any particular feature; therefore, histological examination is essential for establishing a diagnosis. Microscopically, CSM exhibits a solid sheet-like growth pattern, composed of uniformly sized ovoid to spindled or histiocytoid cells with pale eosinophilic syncytial cytoplasm. The nuclei exhibit a vesicular appearance with fine chromatin and small or inconspicuous nucleoli, showing minimal to no atypia. Mitotic activity varies, ranging from 0 to 4 per 10 HPF, with the majority of reported cases showing no mitotic figures. Necrosis and lymphovascular invasion are consistently absent [3].

Immunohistochemically, CSM is characterized by the co-expression of smooth muscle and epithelial markers. From the literature, it can be seen that it is frequently positive for EMA (100%), S-100 protein (87%), and AE1/AE3 (82%), and to a lesser degree for SMA (69%), p63 (55%), and GFAP (37%) [8].

In our case, an additional unique finding was the focal positivity for CD34, which has never been previously observed. This positivity was mostly confined to a central area of the neoplasm and, curiously, it was accompanied by a concomitant loss of SMA expression. Despite this aberrant phenotype, the neoplastic cells still demonstrated a positive staining for S100 and EMA, even stronger compared to the rest of the tumor. Furthermore, the presence of EWSR1 gene rearrangement supported the assumption that this specific area was indeed a part of the neoplasm with a genuine aberrant expression of CD34. To further investigate, additional staining for vascular markers (CD31 and ERG) was performed, which yielded negative results.

CD34 is a transmembrane phosphoglycoprotein initially discovered on hematopoietic stem and progenitor cells. Interestingly, CD34 expression extends beyond mesenchymal stem cells (MSCs) to encompass a wide range of non-hematopoietic cell types, such as muscle satellite cells, corneal keratocytes, interstitial cells, epithelial progenitors, and vascular endothelial progenitors [9]. Its function remains unknown to this day.

In dermatopathology, this marker is used to classify soft tissue tumors and to distinguish CD34+ dermal neoplasms such as Kaposi’s sarcoma (KS) and dermatofibrosarcoma protuberans (DSFP), which are both CD34+, and epithelioid sarcoma, which is often CD34+, from dematofibroma that is typically CD34−. CD34 can be useful in distinguishing solitary fibrous tumors (CD34+) from desmoplastic mesothelioma (CD34−) [10] and in distinguishing hemangiopericytoma (CD34+) from endometrial stromal sarcoma (CD34−) [11].

Fluorescence in situ hybridization (FISH) targeting the EWSR1 gene rearrangement is an important diagnostic tool for CSM. In a series of 38 cases of CSM, Jo et al. [3] reported the detection of EWSR1 rearrangement in 82% of the tested cases. Notably, approximately 90% of these cases showed a gene fusion of EWSR1 with PBX3, representing a peculiar molecular feature among myoepithelial neoplasms, which occurred in most of the CSM cases [4]. Unfortunately, our laboratory currently lacks the additional molecular testing required to demonstrate this specific gene fusion.

The EWSR1 gene exhibits widespread expression across multiple cell types, suggesting its involvement in diverse cellular processes and organ development. EWSR1 plays a crucial role in the epigenetic regulation of gene expression, RNA processing, and cellular signal transduction. Notably, EWSR1 modulates microRNA (miRNA) levels through interaction with Drosha, which subsequently leads to impaired dermal development and dysfunctional autophagy. Despite these significant roles of EWSR1 in various cellular functions, the precise underlying mechanisms remain incompletely understood [12].

PBX3 is a transcription factor belonging to the PBX family, characterized by the presence of a homeodomain. Members of this family play critical roles in early developmental processes as well as certain processes in adulthood. To date, PBX3 is more frequently associated with cancer, and has been reported to be overexpressed in many solid tumors, as well as in several hematological malignancies, where it has a role in promoting cell survival, invasion, and proliferation [13].

The gene fusion EWSR1-PBX3 in CSM is responsible for the generation of a hybrid protein that combines the transactivation domain of EWSR1 with the DNA binding homeodomain of PBX3. It is hypothesized that this fusion protein disrupts the normal regulation of PBX3 target genes [4].

In contrast with the EWSR1 and PBX3 rearrangements shown by CSM, PLAG1 translocations are commonly identified in myoepithelial tumors with duct differentiation. Antonescu et al. reported myoepitheliomas with ductal differentiation and frequent PLAG1 rearrangement, analogous to salivary gland pleomorphic adenomas [14].

The differential diagnosis of CSM includes dermatofibroma (DF), epithelioid sarcoma (ES), early stage juvenile xanthogranuloma (JXG), melanocytic lesions, neurothekeoma (NT), cutaneous myoepithelial carcinoma (CMC) [3,15], and perineurioma.

Histologically, DF manifests as a dermal nodule composed of epithelioid binucleated cells within a fibrovascular stroma; but, unlike CSM, this lesion lacks syncytial architecture [5,15]. ES typically exhibits a combination of epithelioid and spindle cells with cellular atypia and infiltrative growth, often accompanied by satellite nodules [6]. In the early stage, JXG presents as an exophytic lesion commonly found in children that is characterized by the presence of mononuclear or multinuclear lipidized cells. Differently melanocytic lesions, particularly Spitz nevus, are characterized by the presence of melanocytic nests and a syncytial cytoarchitecture. NT is a benign superficial soft tissue tumor occurring usually in women and on the head, neck, and extremities, and it typically presents as a skin-colored papule or nodule [16,17]. Neurothekeoma exhibits a distinctive growth pattern characterized by nested structures, accompanied by varying degrees of a myxoid background. CMC is an extremely rare neoplasm with a poor prognosis which clinically presents as an asymptomatic nodule [18,19] and arises more frequent on limbs. Histologically, it shows trabecular, reticular, nested, or solid growth of variably spindled or epithelioid cells with frequent myxoid or hyalinized stroma. Moderate to severe nuclear atypia, increased mitotic activity, and necrosis are present in variable degrees. Finally, perineurioma may be considered among the differential diagnoses. It is an uncommon mesenchymal neoplasm of the peripheral nerve sheath with perineurial differentiation. Cells typically present with slender nuclei and delicate elongated bipolar cytoplasmic processes [20].

Based on immunohistochemistry, DF cells frequently exhibit binucleation and show positivity for EMA [5,15]; but, unlike syncytial myoepithelioma, this lesion does not demonstrate immunostaining for S100 protein and p63. Similarly to CSM and DF, ES lesions are positive for EMA, but they differ in terms of their positivity for cytokeratins and CD34. Additionally, ES is negative for myoepithelial markers such as S100 protein and GFAP, and frequently exhibits the loss of immunostaining for INI-1 [3,15]. JXG lesions show negativity for EMA, in contrast to CSM, DF, and ES. Cells are also negative for S100 while they are positive for markers associated with histiocytic origin such as CD163 and CD68 [5]. Also, Spitz nevus shows negative staining for EMA and the diagnosis is confirmed by positive immunostaining for Melan-A and HMB-45 [3,5,15]. Similarly to DF, ES, JXG, and perineuroma immunohistochemistry is negative for S100 and typically positive for vimentin, MiTF, CD10, and NKI/C3 [16,17]. CMC lesions are typically positive for EMA and S100, as with CSM, but cells are also positive for AE1/AE3 and negative for CD34 and ERG [18,19]. Finally, perineurioma cells are also positive for EMA, but unlike CSM, cells are negative for the S-100 protein [20].

Focusing on CD34 expression, some authors have reported positivity with CD34 in DF, especially in the acral region of the lesion [21]. Equally, cells share a common CD34 expression in ES and JXG [22,23]. CD34 expression has also been variably observed in perineuriomas, especially in the sclerosing type [24].

Nowadays, because of the absence of specific guidelines, the only treatment option described for CSM is surgical excision. Other options, such as radiotherapy or chemotherapy, have never been reported in the literature. Currently, because of its histopathological features (low mitotic index, absence of cellular atypia, and necrosis) and clinical behavior, it is generally considered as a benign neoplasm [7]. In fact, no cases of disease progression or distant metastasis have been reported in the literature. Only one patient experienced a local recurrence 51 months after surgery, despite the initial excision being performed via a shave biopsy with a positive deep margin [3]. In contrast, some authors believe that histopathologic features appear to be a poor indicator of prognosis because some myoepitheliomas, of other variants than the syncytial one, develop metastasis, although with a benign morphological appearance [25]. In addition, there is a lack of evidence regarding the timing of follow-ups and the potential need for diagnostic investigations.

## 4. Conclusions

CSM is a very rare benign skin tumor with entirely nonspecific clinical manifestations.

Histological examination is essential for establishing a diagnosis of CSM, and fluorescence in situ hybridization (FISH) is an important diagnostic tool. Furthermore, our case demonstrated focal positivity for CD34, which has never been previously observed.

The best treatment option for CSM is surgical excision. Because of the benign clinical behavior of CSM, the proliferation index of the lesion (Ki67 < 5%) and the weak presence of low mitoses, we opted for a close clinical follow-up (every 6 months for the first two years). No disease recurrence was observed in our patient 12 months after surgery; the follow-up still ongoing.

## Data Availability

The data presented in this study are available on request from the corresponding author.
